# Constantly Bombarded with New Drugs: What’s a Cardiologist to Do?

**DOI:** 10.14797/mdcvj.1179

**Published:** 2022-12-06

**Authors:** Albert E. Raizner, James B. Young

**Affiliations:** 1 Houston Methodist DeBakey Heart & Vascular Center, Houston Methodist, Houston, Texas, US; 2 Cleveland Clinic Lerner College of Medicine of Case Western Reserve University, Cleveland, Ohio, US

**Keywords:** cardiovascular therapeutics, guideline-directed therapies, empagliflozin, sodium-glucose cotransporter-2 (SGLT2) inhibitor

## Abstract

Every physician encounters a barrage of direct-to-consumer and direct-to-physician advertising and is faced with the daunting task of deciding which drugs to add to their clinical armamentarium and how and when to add them. The purpose of this Points to Remember is to present a commonsense approach to incorporating newer drugs, or perhaps new indications for older drugs, into our clinical practice. To illustrate these points, this article focuses on a single drug, empagliflozin, a sodium-glucose cotransporter-2 (SGLT-2) inhibitor that has been highly marketed in lay and medical media and hence has been incorporated into professional society treatment guidelines.

This issue of the *Methodist DeBakey Cardiovascular Journal* focuses on pharmacology in cardiology practice. The major emphasis is to look at new drugs that may have been recently released, drugs for which new indications have surfaced, and drugs that are not yet approved but are in the pipeline for approval. The dilemma facing clinicians is how to incorporate new drugs or new indications for old drugs into our everyday practice.

New drugs, and new indications for established drugs, come to our attention via a variety of mechanisms. Of course, the classic dissemination of drug information is through medical literature, particularly peer reviewed articles, on subjects that are within our clinical realm. Also important are therapeutic guidelines developed by our professional societies, such as the recent American College of Cardiology, American Heart Association, and Heart Failure Society of America 2022 Guideline for the Management of Heart Failure.^1^ The problem with guideline development, however, is disagreement about the evidence chosen to support recommendations and the length of time it takes to create, disseminate, and actualize what we have come to call “guideline-directed therapies.”

In recent years, this approach to newer therapeutics has given way to a more subtle solicitation through internet-based medical articles with commercial embeds. An even more surreptitious and troublesome approach by the pharmaceutical industry has been that of direct advertising to patients (and vicariously to physicians) via television, radio, and the digital medium, an approach particularly prominent in the United States (US). In fact, it is estimated that the US pharmaceutical industry spent $6.58 billion dollars in 2020 on direct-to-consumer advertising. This tactic has proven to be highly effective because advertising prompts patients to ask their physicians, “Would I benefit from drug X for my condition?” Furthermore, it is not unusual for busy physicians who do not have time to peruse their medical journals to first become acquainted with a drug via these advertisements.

Given this barrage of direct-to-consumer and direct-to-physician advertising, every physician is faced with the daunting task of deciding which drugs to add to our clinical armamentarium and how and when to add them. The purpose of this Points to Remember column is to present a commonsense approach to incorporating newer drugs, or perhaps new indications for older drugs, into our clinical practice.

To illustrate these points, we will focus on a single drug, empagliflozin, a sodium-glucose cotransporter-2 (SGLT2) inhibitor, which has been highly marketed in lay and medical media, has been incorporated into professional society treatment guidelines, and is discussed in more detail in this issue (see “The Evolution of Glucocentric Drugs in Cardiovascular Disease Protection and Heart Failure” by Talha, Butler et al.).

## Points to Remember about Newer Cardiovascular Therapeutics

1. Regardless of how a drug or new indication comes to our attention, ***our first decision should be whether or not it is relevant to our clinical practice***. For example, empagliflozin was originally approved by the US Food and Drug Administration in 2014 as a supplement to diet and exercise to improve glucose control in adults with type 2 diabetes. As such, it initially received little interest among cardiologists. However, in 2015, the same drug was shown to improve cardiovascular outcomes in diabetics at high risk for cardiac events.^2^
2. Once it is suggested that a drug may have some pertinence to your clinical practice, ***it is necessary to learn more about it before prescribing it to a patient***. This is where the Internet becomes your ally. For most drugs, the pivotal studies that were used to gain their approval or authorize a new indication should be the first source to review. Upon closer review, these pivotal studies often focus on a relatively narrow group of patients with very defined disease specifications. In the case of empagliflozin, the EMPA-REG OUTCOME (Empagliflozin Cardiovascular Outcome Event) trial demonstrated in type 2 diabetes patients with established cardiovascular disease that empagliflozin significantly reduced CV deaths, all-cause mortality, and hospitalizations for heart failure compared with placebo.^1^ The findings were irrespective of its antihyperglycemic effect. As such, it fell into the potential armamentarium of cardiology.
3. Before prescribing the drug, ***the side effects and potential drug interactions should be reviewed***. Very often, the real-world experience of a drug does not reflect the same safety profile that the pivotal studies described. Empagliflozin, for example, is contraindicated in patients with severe renal impairment, end-stage renal disease, or who are on dialysis and, among other concerns, may increase the risk of diabetic ketoacidosis in patients with type 1 diabetes.
4. Once you feel comfortable understanding a drug, its clinical indications, and its usefulness as well as potential side effects, ***an appropriate patient or patients should be identified***. Early and frequent follow-up would be very helpful both in learning more about the effects of the drug and its safety in patients.
5. Despite our desire to provide the best pharmacotherapy for our patients, the cost, particularly of new drugs, often enters into our ability to provide optimal treatment. ***Consideration of a cost-to-benefit ratio*** is an unfortunate reality for many patients with limited resources or inadequate insurance benefits.
6. In some cases, you may find that your patients’ experiences with a drug are not quite the same as the pivotal clinical trial data would suggest. This is not very unusual in that clinical trials utilize the drug in a large population of patients, some of whom reacted as presented, but there are always some who did not. Furthermore, the real-world use of a drug (or device, for that matter) reveals issues not heretofore recognized. As a clinician, ***your personal experience becomes the bottom line in deciding on the ultimate clinical utility of a drug in your practice***.
